# Japan’s voluntary lockdown

**DOI:** 10.1371/journal.pone.0252468

**Published:** 2021-06-10

**Authors:** Tsutomu Watanabe, Tomoyoshi Yabu

**Affiliations:** 1 Graduate School of Economics, University of Tokyo, Bunkyo, Tokyo, Japan; 2 Faculty of Business and Commerce, Keio University, Minato, Tokyo, Japan; Kobe University, JAPAN

## Abstract

Japan’s government has taken a number of measures, including declaring a state of emergency, to combat the spread COVID-19. We examine the mechanisms through which the government’s policies have led to changes in people’s behavior. Using smartphone location data, we construct a daily prefecture-level stay-at-home measure to identify the following two effects: (1) the effect that citizens refrained from going out in line with the government’s request, and (2) the effect that government announcements reinforced awareness with regard to the seriousness of the pandemic and people voluntarily refrained from going out. Our main findings are as follows. First, the declaration of the state of emergency reduced the number of people leaving their homes by 8.5% through the first channel, which is of the same order of magnitude as the estimates obtained for lockdowns in the United States. Second, a 1% increase in new infections in a prefecture reduces people’s outings in that prefecture by 0.027%. Third, the government’s requests are responsible for about one quarter of the decrease in outings in Tokyo, while the remaining three quarters are the result of citizens obtaining new information through government announcements and the daily release of the number of infections. The findings suggest that what mattered for containing the spread of COVID-19 was not strong, legally binding measures but the provision of appropriate information that encouraged people to change their behavior.

## 1 Introduction

In response to the spread of COVID-19, the Japanese government declared a state of emergency on April 7 for seven prefectures, including Tokyo, and on April 16 expanded the state of emergency to all 47 prefectures. Prime Minister Abe called on citizens to reduce social interaction by at least 70% and, if possible, by 80% by refraining from going out. In response to these government requests, people restrained from going out. For example, in March, the share of people in Tokyo leaving their homes was down by 18% compared to January before the spread of COVID-19, and by April 26, during the state of emergency, the share had dropped as much as 64%. As a result of people refraining from leaving their homes, the number of daily new infections in Tokyo fell from 209 at the peak to two on May 23, and the state of emergency was lifted on May 25.

Unlike the lockdowns in China, the United States, and European countries such as Italy, restrictions during Japan’s state of emergency had no legal binding force. There were no penalties such as fines or arrests for leaving the house during the state of emergency. The police did not warn anyone who was out on the streets. The situation in Japan was one of a “voluntary lockdown.” Looking at the “Government Response Stringency Index”—a composite measure of nine response indicators published by the University of Oxford’s Blavatnik School of Government—shows that the value for Japan of 47.22 at the end of April during the state of emergency was considerably smaller than those for France (87.96), the United States (72.69), the United Kingdom (75.93), Germany (76.85), Italy (93.52), and Canada (72.69). Instead, the value for Japan was essentially on the same level as that for Sweden (46.30). Looking at individual indicators, the status for “Restrictions on public gatherings” was “No restrictions” and that for “Closures of public transport” was “No measures,” which is quite different from other countries. Similarly, with regard to “Stay-at-home requirements,” restrictions in Japan were weaker than in other countries: while in Japan people were “recommended” to stay at home, in the United States and various European countries they were “Required not to leave the house with exceptions.”

The fact that the behavior of people in Japan changed even though the country only took measures that were not legally binding suggests that the (legally binding) lockdowns in other countries were not the only reason people changed their behavior. The aim of this study is to clarify the mechanisms by which the non-legally binding policies of the Japanese government led people to change their behavior such as refraining from going out.

In this study, we focus on the following two channels through which the Japanese government’s measures to prevent the spread of COVID-19 changed people’s behavior. The first channel is the “intervention effect.” This refers to changes in people’s behavior as a result of obeying government orders and requests to refrain from leaving their homes. In the case of first Wuhan in China and then the United States and Europe, this took the form of severe lockdowns, in which the government used legal powers to deprive people of their freedom of movement. In Japan’s case, the state of emergency was not legally binding and, if anything, was very limited, so that it is appropriate to regard it as a “request” from the government. On the other hand, the closure of schools by the Japanese government had a compulsory aspect.

The second channel is the “information effect.” Generally, when a government implements a policy on something, it can be assumed that it makes its decisions based on various types of information gathered before reaching the decision. Therefore, government decisions provide the public with information about the current situation. This is the signaling effect of government measures. Applying this to measures such as the state of emergency declaration, it can be thought that the public obtained new information on the status of infections through the government’s announcements. In developed countries, including Japan, details on infections are generally not disclosed to the public to protect the privacy of those infected. This means that governments have considerably more information than is in the public domain (or at least this is what many people believe), so that government actions have a strong signaling effect.

To examine the role that these two channels played in affecting people’s behavior, we use smartphone location data to construct a daily prefecture-level measure showing the degree to which people stayed at home. We then construct panel data to examine the effect of the two major government measures to contain infections—the declaration of the state of emergency and the closure of schools—on the stay-at-home measure. Importantly, in doing so, we distinguish between the intervention effect and the information effect. We do so by utilizing the fact that the timing of the start and the end of the state of emergency and of school closures differed across prefectures.

There already is a considerable body of research on the economic impact of COVID-19, and the number of studies is increasing rapidly. Against this background, the present study is most closely related to the following three areas of research. The first is research on changes in behavior and the reduction in outings using smartphone location data. Examples of studies in this area include [1–5].

The second related area is studies on the impact of lockdown policies in the United States on people’s movement and behavior. Examples include the studies by [1, 5–9]. The main interest of the present study is why the Japanese government’s countermeasures against COVID-19 brought about changes in behavior even though they were not legally binding, and what is interesting in this regard is that a number of studies using U.S. data, including [7, 9, 10], highlight that the lockdowns imposed by governments account for only a limited part of changes in people’s behavior in the United States. A study using data for a country other than the United States is that by [11], who compare Denmark, where the government used legal interventions on outings and economic activities to halt the spread of infections, and Sweden, where the government did not make such interventions. They show that the decline in economic activity was not significantly different and argue therefore government interventions were not a major cause of the economic contraction.

The third area to which our study is related is research on the effects of lockdown policies in Asia. Focusing on China and using mobile phone location data, ref [12] use difference-in-differences estimation to examine how the movement of people changed following the lockdown in Wuhan. Focusing on South Korea, ref [13] compared the Daegu-Gyeongbuk area, where infections were especially prevalent, with other areas in South Korea using difference-in-differences estimation.

## 2 Outbreak of COVID-19 and policy responses in Japan

The first reported case of a COVID-19 infection in Japan—of a man who had traveled to Wuhan, China—was on January 16, 2020. Then, on February 5, 10 passengers of a cruise ship docked at Yokohama Port were confirmed to have caught the virus. The first death in Japan was reported on February 10. In response to the spread of infections, the government on February 27 requested elementary, junior high, and high schools nationwide to temporarily close, and on March 24 decided to postpone the Tokyo Olympic Games scheduled for the summer of 2020. Furthermore, on April 7, a state of emergency was declared for seven prefectures including Tokyo, and on April 16, this was expanded to all prefectures.

**Fig 1:**
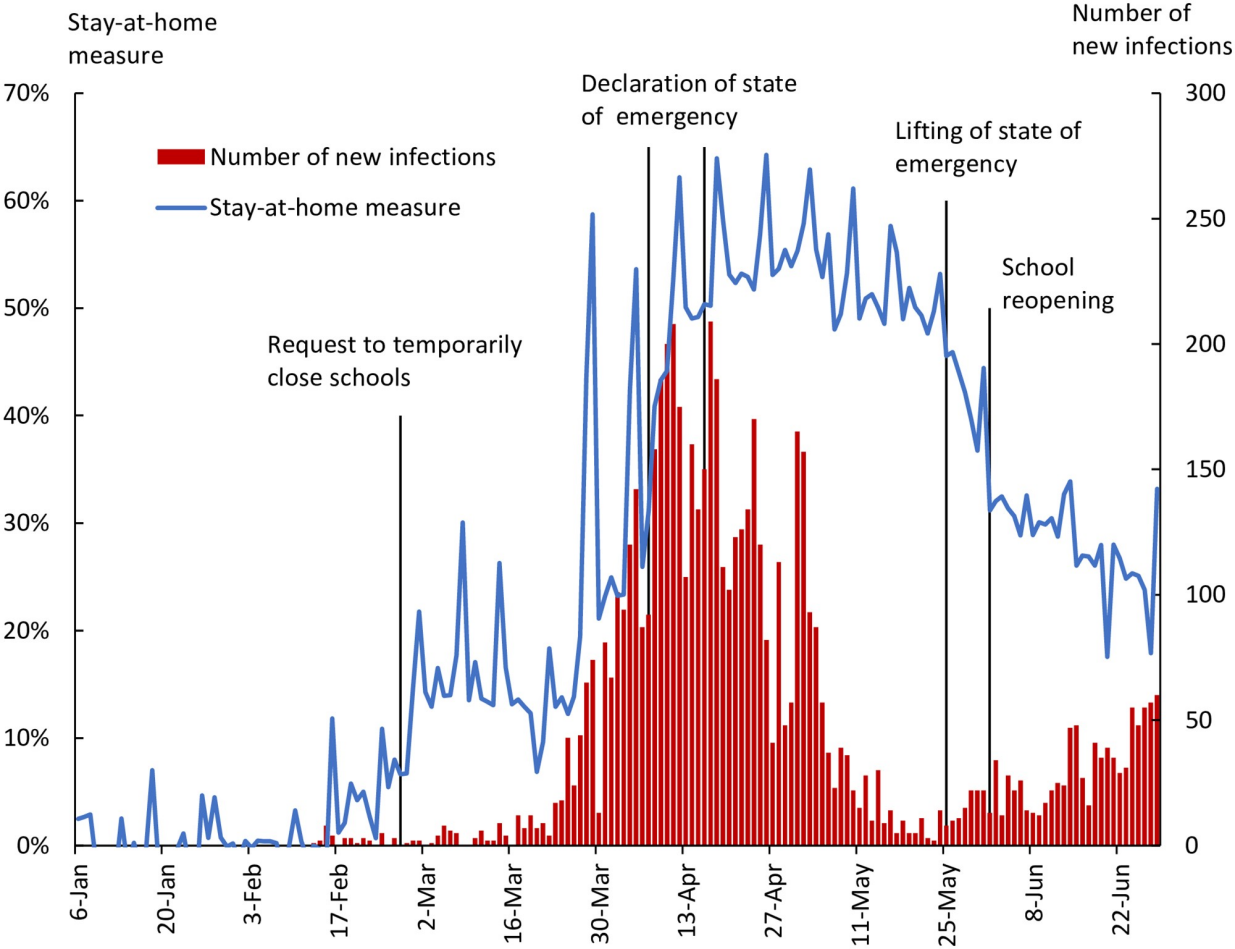
Stay-at-home measure and number of new infections, Tokyo.

Fig 1 shows the number of daily new infections in Tokyo, represented by the orange bars. The number of new infections increased rapidly in late March, exceeding 100 on March 17 and exceeding 200 on April 10. With the declaration of the state of emergency, the number of new infections decreased and fell to almost zero in mid-May. However, the number of new infections started to increase again in the second half of May.

The blue line in Fig 1 is the stay-at-home measure created using mobile phone location data. The line shows the extent to which Tokyo residents refrained from leaving their home compared to January 2020, before the pandemic. The Figure indicates that as the number of new infections increased, people increasingly stayed at home. This suggests the possibility that people updated their information on infections based on the number of new infections announced daily by the Tokyo governor and changed their behavior to avoid infection. Moreover, the stay-at-home measure jumped following the request for the temporary closure of schools on February 27 and the declaration of the state of emergency on April 7, showing that the government’s measures changed people’s behavior. Interestingly, the stay-at-home measure also increased on April 16, when the state of emergency was expanded to all prefectures. Specifically, the stay-at-home measure increased from 0.50 in the week of April 9-15 to 0.55 in the week of April 16-22. The state of emergency for Tokyo had already been declared on April 7, and the measure on April 16 should not have directly affected the residents of Tokyo. However, it is possible that although the measures targeted other prefectures, Tokyo residents obtained new information on the spread of infections from the government’s announcement of the measure. Another point to note is that the stay-at-home measure remained at a high level of over 20% and thus higher than in January despite the fact that the state of emergency was lifted from late May to early June and schools were reopened. If the government’s request to refrain from leaving home was the main reason for the change in people’s behavior, the stay-at-home measure should have dropped to its original level as the request was lifted. That has not been the case, suggesting that a significant part of the change in people’s behavior is voluntary.

**Fig 2:**
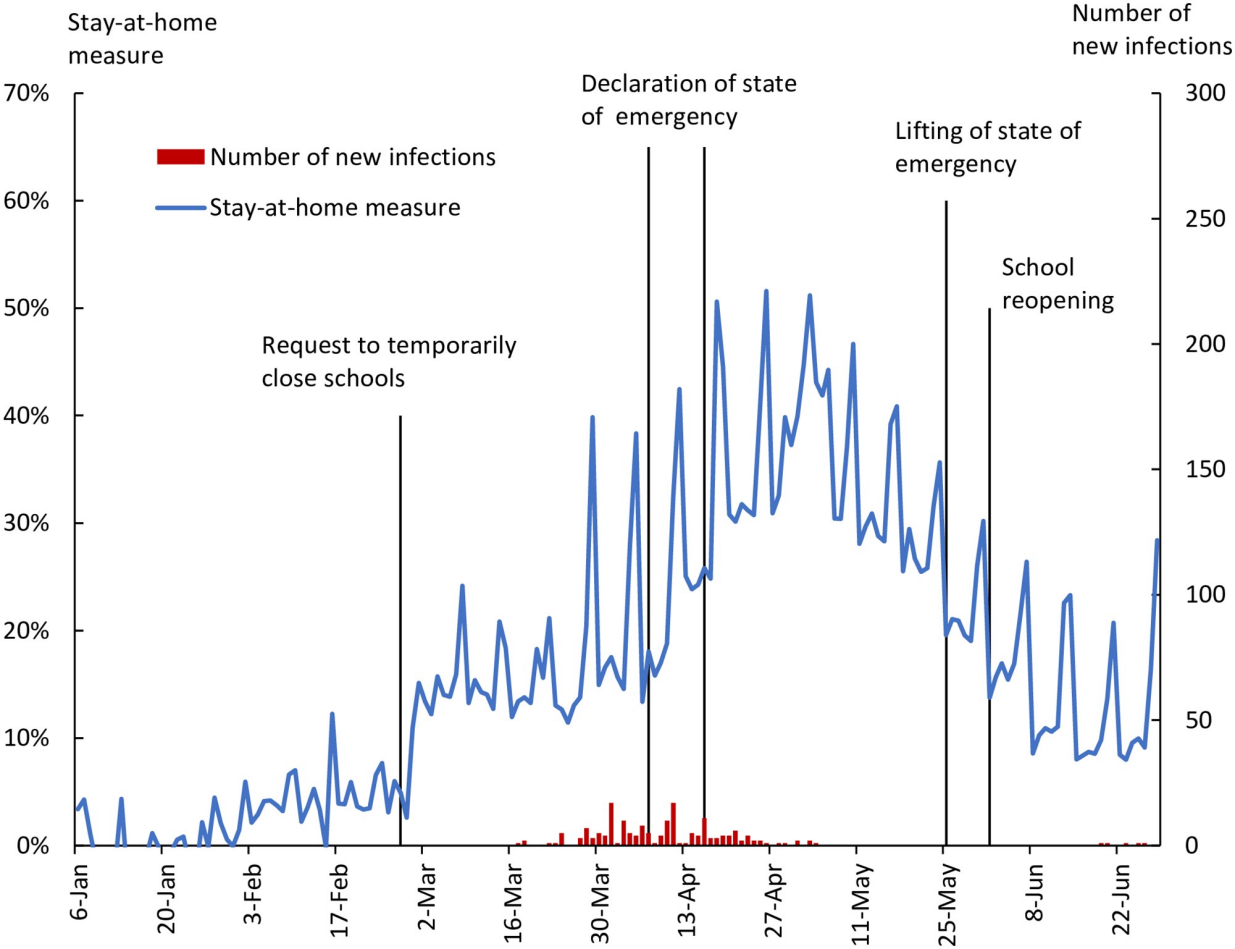
Stay-at-home measure and number of new infections, Ibaraki.

While Tokyo is the prefecture with the highest number of infections in Japan, some prefectures have had zero or very few infections. Fig 2 shows an example of a prefecture with a small number of infections. Specifically, it shows Ibaraki prefecture, which is located northeast of Tokyo. By the end of June, the total number of infections in Ibaraki prefecture was 179, which is only 3% of the number for Tokyo. The stay-at-home measure for Ibaraki jumped immediately after the request for schools to close on February 27 and the declaration of the state of emergency on April 16. This indicates that the government’s request to refrain from leaving home had a certain effect even in areas with few infections such as Ibaraki prefecture. In addition, the stay-at-home measure for Ibaraki prefecture also shows a jump on April 7, when the state of emergency was declared for seven prefectures including Tokyo, but not Ibaraki itself. This suggests that residents of Ibaraki prefecture have been paying close attention to the situation in areas with high infections and have changed their behavior based on this.

## 3 Methodology

We construct and examine the stay-at-home measure for each of the 47 prefectures of Japan for the period from January 6 to June 28, 2020. Using this panel data, we identify the following.

First, we identify the “intervention effect” and the “information effect.” Government policies such as the declaration of the state of emergency and school closures occurred at different times across prefectures, and by using these differences in timing, it is possible to determine which of the two effects was responsible for changes in people’s behavior. For example, a state of emergency was declared in Tokyo on April 7, but at that time no state of emergency was declared for Tochigi prefecture, which is located 100km north of Tokyo, about an hour away on the bullet train. A state of emergency was declared in Tochigi on April 16. Therefore, there was no intervention effect in Tochigi prefecture from April 7 to 15. However, people in Tochigi prefecture were aware that the state of emergency had been declared in Tokyo, so there was an information effect, and the stay-at-home measure rose accordingly. On the other hand, people in Tokyo refrained from leaving home due to both the intervention and information effects, and the stay-at-home measure rose. Therefore, assuming that people in Tokyo and Tochigi had the same information about infections and there was no difference in the information effect between the two prefectures, the intervention effect can be extracted by observing the difference in the stay-at-home measure between the two prefectures. As for the declaration of the state of emergency, not only did the time when it was declared differ across prefectures, but the lifting also occurred in three waves, so that differences in the timing of the lifting of the state of emergency can also be used to identify the intervention effect. Similarly, with regard to school closures, the timing of when school closures were lifted varies widely across prefectures, and this can also be used to identify the intervention effect. Meanwhile, since all measures against COVID-19 are carried out at the prefectural level, there are few differences across smaller administrative units within the same prefecture such as municipalities.

The second identification we carry out is to examine whether changes in people’s behavior depend on infections in their surroundings or in Japan as a whole. As seen in Fig 2, even in prefectures with a small number of new infections, people refrained from leaving home. This suggests that people may be making decisions about refraining from leaving home in response to the number of infections nationwide, not the number of infections in the prefecture. Since infections are concentrated in metropolitan areas such as Tokyo, the number of infections in such metropolitan prefectures and the number of infections nationwide are strongly correlated. However, in other prefectures, the number of infections in that prefecture and the nationwide number are only weakly correlated. Using this property, we can estimate to what extent the change in people’s behavior is due to the number of infections within a prefecture or the number of infections nationwide.

The empirical approach used in this study is as follows. The stay-at-home measure at time *t* in prefecture *i* is denoted by *y*_*it*_. The number of new infections at time *t* in prefecture *i* is denoted by ${\tilde{x}}_{it}$. The number of new infections nationwide is denoted by ${\tilde{x}}_{t}$. The distribution of the number of new infections is skewed to the right because the number of new infections is much larger in a small number of prefectures such as Tokyo than most other prefectures. While many existing studies use logarithms to cope with such highly skewed distributions, for some of the prefectures in Japan the number of new infections is zero on some days, so that we cannot take logarithms. Following Goolsbee and Syverson (2020), we transformed ${\tilde{x}}_{it}$ and ${\tilde{x}}_{t}$ using the inverse hyperbolic sine. Specifically, we define $x_{it}\equiv \ln\left({\tilde{x}}_{it}+\sqrt{{\tilde{x}}_{it}^{2}+1}\right)$ and $x_{t}\equiv \ln\left({\tilde{x}}_{t}+\sqrt{{\tilde{x}}_{t}^{2}+1}\right)$. The estimation equation used in this study is as follows:

$$\begin{array}{c} y_{it}=\mu_{i}+\underset{\text{Intervention}\;\text{effect}}{\underset{︸}{\alpha_{0}D_{it}\left(\text{Emergency}\;\text{declaration}\right)+\beta_{0}D_{it}\left(\text{School}\;\text{closure}\right)}}\\ +\underset{\text{Information}\;\text{effect}}{\underset{︸}{\sum_{k}\alpha_{k}A_{t}\left(E_{k}\right)+\sum_{k}\beta_{k}A_{t}\left(C_{k}\right)+\gamma_{1}x_{it}+\gamma_{2}x_{t}}}+\epsilon_{it}\; \end{array}$$ (1)

where *μ*_*i*_ represents the effect unique to prefecture *i*. *D*_*it*_(*Emergencydeclaration*) is a dummy variable that takes 1 when the state of emergency is active at time *t* in prefecture *i*, and 0 otherwise. Similarly, *D*_*it*_(*Schoolclosure*) is a dummy variable that takes 1 when schools are closed at time *t* in prefecture *i*, and 0 otherwise. *A*_*t*_(*E*_*k*_) represents the government’s *k*th announcement regarding the state of emergency. It is a dummy variable that takes 1 in all prefectures after the *k*th announcement. Similarly, *A*_*t*_(*C*_*k*_) is a dummy variable that represents the government’s announcements with regard to school closures.

In Eq (1), it was assumed that the source of people’s information on infections was government policy announcements and the number of new infections. However, people may be able to obtain information about infections by other means. To take this into account, we also conduct estimations using the following equation:

$$\begin{array}{c} y_{it}=\mu_{i}+\underset{\text{Intervention}\;\text{effect}}{\underset{︸}{\alpha_{0}D_{it}\left(\text{Emergency}\;\text{declaration}\right)+\beta_{0}D_{it}\left(\text{School}\;\text{closure}\right)}}\\ +\underset{\text{Information}\;\text{effect}}{\underset{︸}{\lambda_{t}+\gamma_{1}x_{it}}}+\epsilon_{it}\;\;\;\;\;\; \end{array}$$ (2)

The specification of the intervention effect is the same as in Eq (1). On the other hand, regarding the information effect, government policy announcements are expressed as a time fixed effect, which is denoted by *λ*_*t*_ in Eq (2), since such announcements provide the same information to the residents of all prefectures. For the same reason, the number of new infections nationwide is also expressed as a time fixed effect. However, the time fixed effect differs from Eq (1) in that it also contains information other than about infections.

## 4 Data

### 4.1 The stay-at-home measure

For our location data, we use the “Mobile Spatial Statistics” provided by DoCoMo Insight Marketing. The Mobile Spatial Statistics provide location records of about 78 million DoCoMo mobile phones at 10-minute intervals. Specifically, the mobile phone base stations in a particular area know which mobile phones are in the area. Based on this information, and dividing Japan into a mesh of 500m × 500m squares, DoCoMo compiles and publishes data on how many mobile phones are in a certain mesh element at a particular time (in 10-minute intervals), together with information on the age and sex of the owners of those mobile phones as well as the municipality they live in.

Using this data, we construct our stay-at-home measure in the following two steps. The first step consists of the detection of residential areas. For a certain mesh element, we count the average number of people in the time from midnight to 5am and take this as the nighttime population of that mesh element. Similarly, we count the number of people in the time from 9am to 5pm and take this as the daytime population of that mesh element. Based on this information, we identify an area as residential if the daytime population is smaller than the nighttime population multiplied by a parameter prespecified to be in the range from 0 to 1. We set the parameter to 0.8 but confirm that the results are essentially the same when we set the parameter to 0.7 or 0.9.

The second step is the calculation of the ratio of those leaving their homes. For mesh elements that in the first step were identified as residential areas, we calculate the number of people leaving home by counting the nighttime population and daytime population on a certain day and subtracting the daytime population from the nighttime population. Next, for each prefecture, we calculate the number of people leaving their homes each day by aggregating the number of people that have left their homes in each mesh element.

Finally, we take the number of persons leaving their homes in January 2020 (January 6 to 31), i.e., before the outbreak of COVID-19, as the number of persons leaving their homes during normal times, and then calculate for each prefecture and day the percentage difference from the number of people leaving their homes during normal times. We use the deviation rate multiplied by −1 as the stay-at-home measure. See [14] for details of the calculation procedure for the stay-at-home measure.

Note that our stay-at-home measure is constructed based on cellular base station data rather than GPS data. If we were interested in how many people are in a particular commercial area, such as stores, stations, or parks, GPS data would provide useful and reliable information, since mobile apps requiring a GPS connection, such as Google Maps, are typically on in such commercial areas. However, GPS data may not be that useful when users are at home and therefore do not use location services frequently. In contrast, cellular base station data continues to provide reliable information on users’ location even when they are at home as mobile phones stay connected to the nearest cellular base station. Thus, cellular base station data is more suitable for our analysis, given that the focus of this study is the extent to which people choose to stay at home in response to the outbreak of the pandemic as well as governments’ interventions.

**Fig 3:**
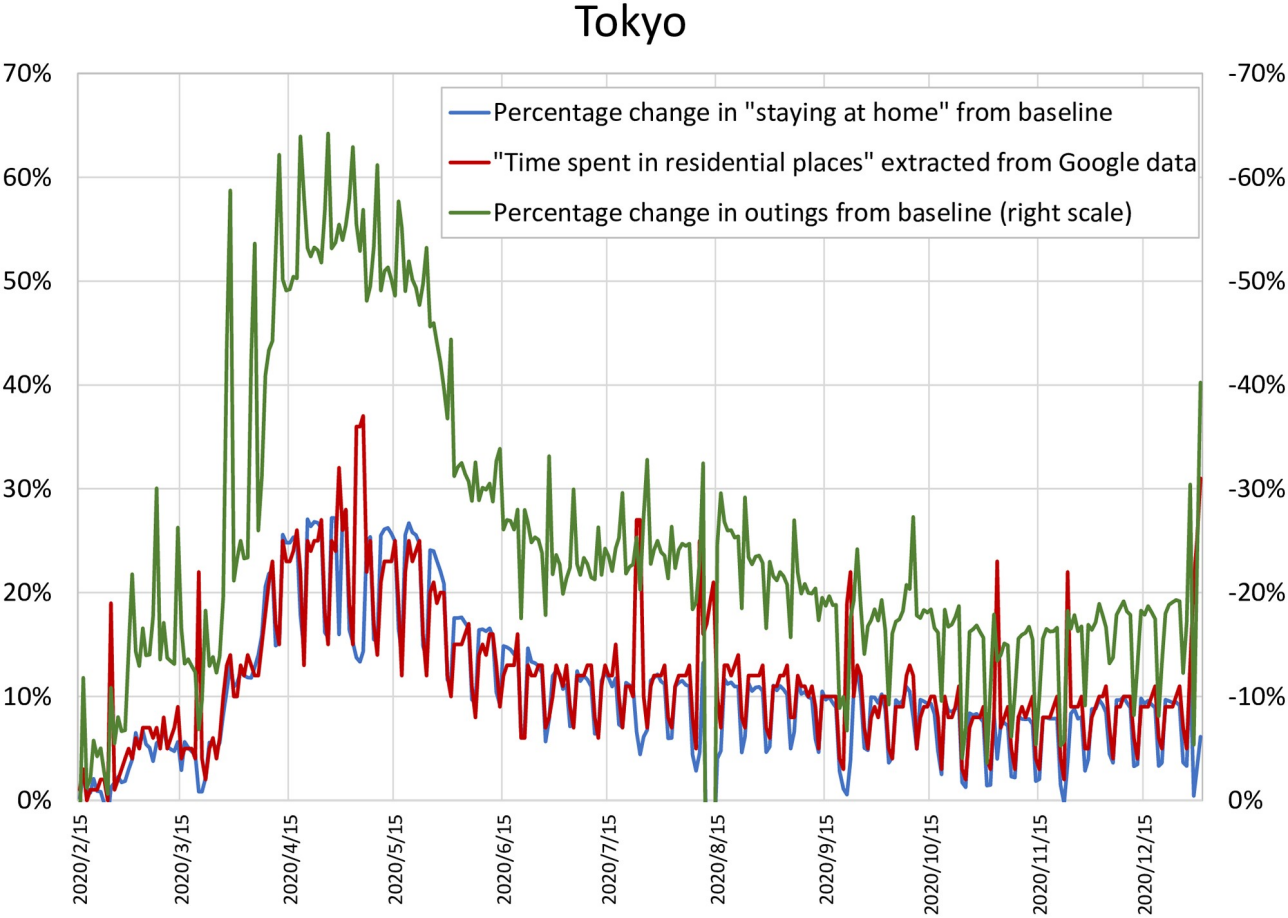
Our Measures and Google’s mobility measure. The blue line shows the percentage change in the level of outings from the corresponding value at the baseline, where the level of outings is defined as the difference between the nighttime and the daytime population. The green line shows the percentage change in the extent to which people stayed at home, which is defined as the daytime population, from the corresponding value at the baseline. Both are constructed from the DoCoMo data. The red line shows the measure of “time spent in residential places” extracted from Google’s Community Mobility Reports.

**Fig 4:**
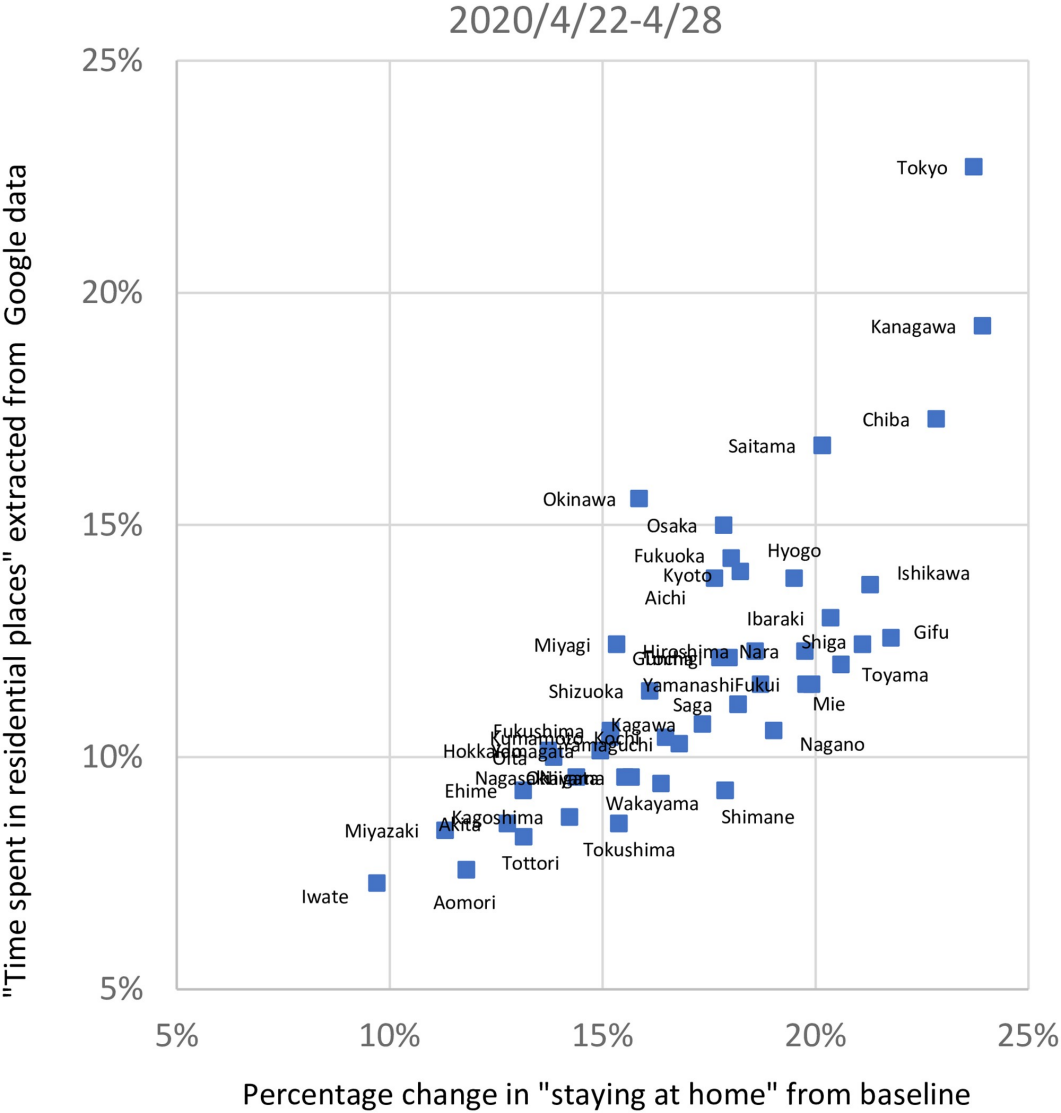
Cross-prefecture comparison. The horizontal axis shows the percentage change in the level of outings from the corresponding value at the baseline, where the level of outings is defined as the difference between the nighttime and the daytime population. The vertical axis shows the measure of “time spent in residential places” extracted from Google’s Community Mobility Reports. Both are the average of daily observations from April 22, 2020 to April 28, 2020.

To provide a sense of how our data compare with the widely used Google mobility data, Figs 3 and 4 compare two mobility measures constructed from the DoCoMo data with a prefecture-level measure of “time spent in residential places” extracted from Google’s Community Mobility Reports (https://www.google.com/covid19/mobility/). Specifically, we present two different mobility measures constructed from the DoCoMo data: (1) the percentage change in the level of outings from the corresponding value at the baseline, where the level of outings is defined as the difference between the nighttime and the daytime population, and (2) the percentage change in the extent to which people stayed at home, which is defined as the daytime population, from the corresponding value at the baseline. The first is identical to the stay-at-home measure that we use in our empirical exercise in the next section, except that it has the opposite sign. Given that Japan’s central and local governments asked people to reduce the level of outings by 80%, this measure is more suitable for measuring the effectiveness of the state of emergency declaration. Meanwhile, the second is conceptually closer to Google’s measure, and Fig 3, focusing on Tokyo, shows that it also moves quite closely with Google’s measure, suggesting a close similarity between these two datasets. Turning to the first measure, Fig 3 shows that this exhibits essentially similar fluctuations over time as Google’s measure, again indicating a close similarity between the datasets; however, because of the way the measure is constructed, there is a gap between the two, which is not constant over time, and there is a larger gap in April and May 2020 than in later period. As a further comparison of the DoCoMo data with the Google data, Fig 4 shows the cross-prefecture variation in the second measure and Google’s measure for the week starting April 22, 2020. The Figure indicates that there is a positive correlation between the two, although our measure tends to be greater than Google’s measure for some prefectures, especially prefectures with small populations. Overall, the exercises in Figs 3 and 4 suggest that the data underlying the mobility measure used in the remainder of the study and the Google mobility data paint a similar picture of mobility trends.

### 4.2 Number of new infections

The central government and prefectures announce the number of new infections daily. The date of infection is the day when a doctor confirms that a person’s polymerase chain reaction (PCR) test was positive (test result date). We use figures from the database constructed and published by JAG Japan Co., Ltd. The number of new infections varies greatly depending on the day of the week. In the analysis here, we assume that people make their decision on whether to leave their homes or not based on the trend in new infections over the preceding week, and we therefore use the moving average over the preceding week including the day in question. Note that throughout this study we do *not* use per capita measures since the number of new infections announced by the government every day is not on a per capita basis but is a raw number, and it is this raw number that determines the extent to which people stay at home.

### 4.3 Government’s measures against the spread of COVID-19

#### 4.3.1 School closures

**Fig 5:**
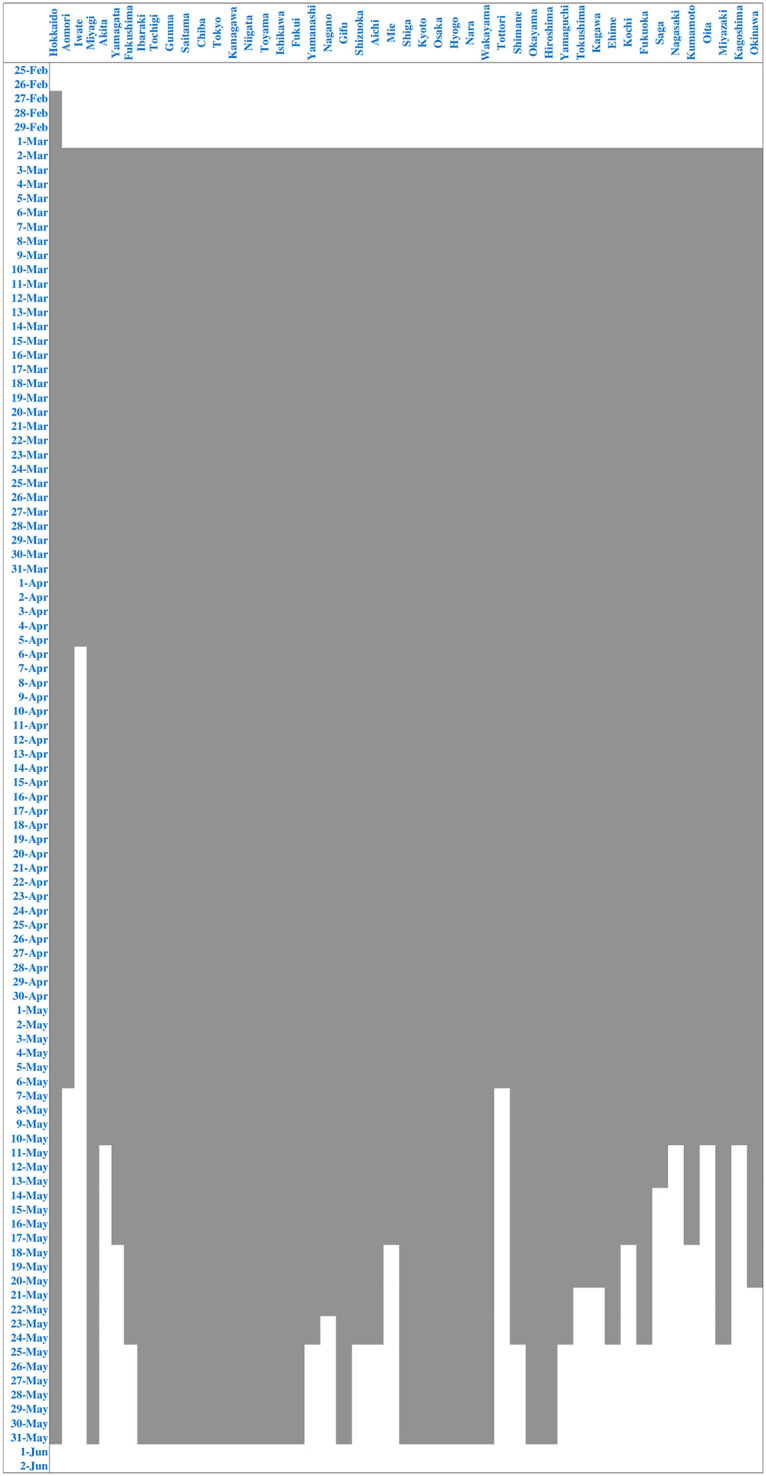
Start and end of school closures.

On February 27, the government requested all elementary schools, junior high schools, high schools, and special needs schools to be closed from March 2 onwards. In response to this, all prefectures except Hokkaido closed schools from March 2. We constructed the following two dummy variables for school closures. *School closure* is a dummy variable that takes 1 during the period schools were closed in a particular prefecture, and 0 otherwise. The date of the reopening of schools varies widely across prefectures: the earliest date was April 6, while the latest date was June 1. The start and end dates for school closures for each prefecture are shown in Fig 5. The second dummy variable, *School Closure Announcement*, represents the announcement of the government’s request for schools to close and is set to 1 for all prefectures except Hokkaido from the day after February 27, when the government made the request.

#### 4.3.2 State of emergency

**Fig 6:**
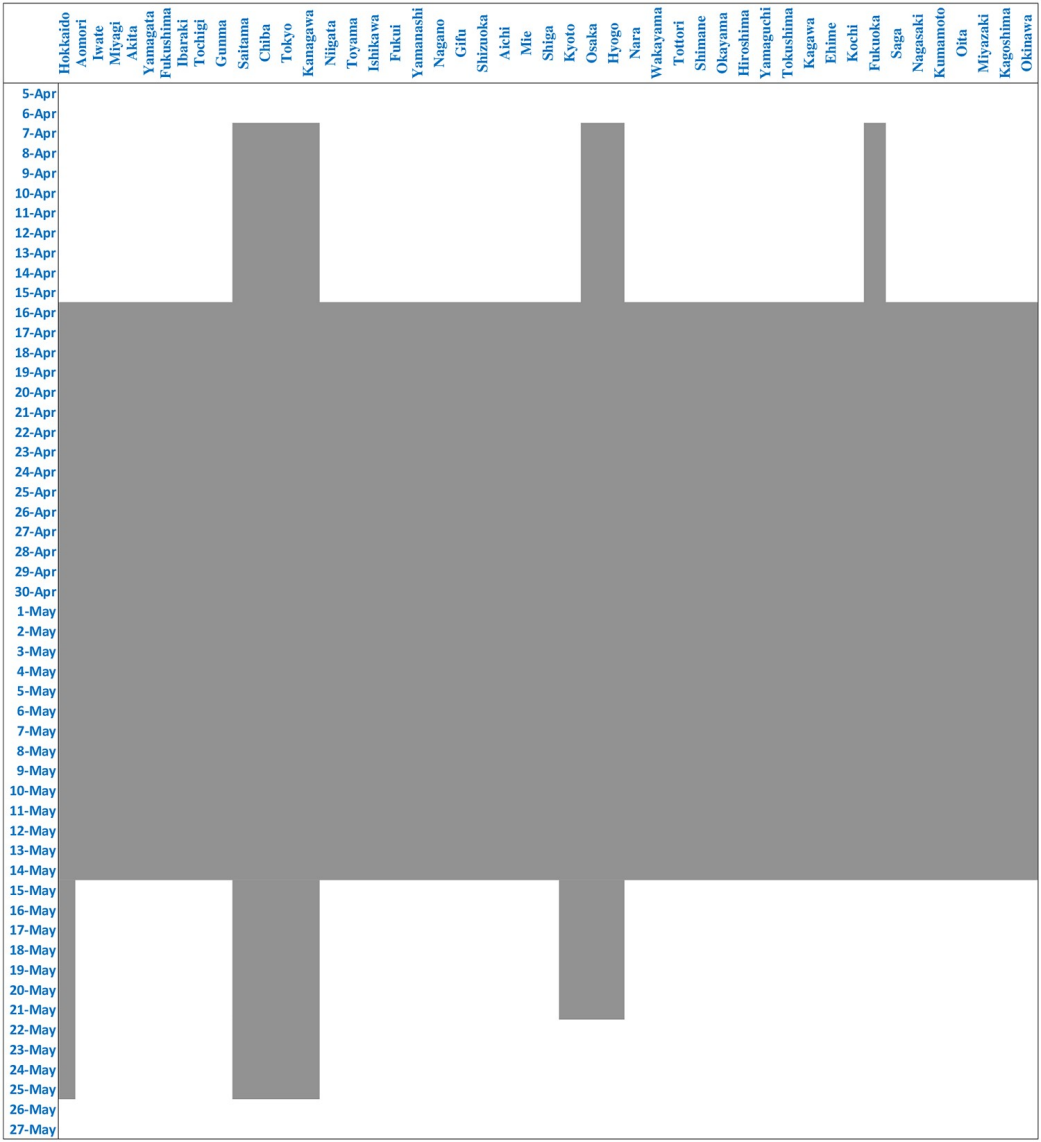
Start and end of state of emergency.

The government declared a state of emergency for seven prefectures on April 7, and expanded the state of emergency to all prefectures on April 16. The state of emergency was lifted in 39 prefectures with few infections on May 14, in three more prefectures on May 21, and finally in the remaining five prefectures including Tokyo on May 25. We construct two types of dummies for the state of emergency. The first, *State of Emergency*, is a dummy that takes 1 when the state of emergency is active in a particular prefecture, and 0 otherwise. The start and end dates of the state of emergency for each prefecture are shown in Fig 6. The second type of dummies, *State of Emergency Announcement*, represents the government’s announcements regarding the start and end of the state of emergency, and takes 1 for all prefectures from the day after the announcement.

## 5 Results

### 5.1 Regression results

We used the stay-at-home measure, expressed in percent, as the dependent variable and conducted the estimations using a fixed effects regression model. The stay-at-home measure is created from smartphone data for each prefecture; however, since the number of smartphones differs across prefectures, the accuracy of the stay-at-home measure also differs across prefectures. To take this into account, we use weighted least-squares estimation, using the number of smartphones in the residential areas of each prefecture as weights. The results are presented in Table 1. In specification (1), the estimation was performed using the *School Closure* dummy and the *State of Emergency* dummy as explanatory variables, and adding the *Rain* and *Weekend/Holiday* dummies as other explanatory variables. The estimation results indicate that both school closures and the state of emergency had a significant effect, with the former raising the stay-at-home measure by 12 percentage points and the latter raising it by 20 percentage points.

**Table 1:** Regression results.

	(1)	(2)	(3)	(4)	(5)
School Closure (SC)	12.119*** (0.269)	3.413*** (0.529)	9.325*** (0.523)	8.398*** (1.169)	4.797*** (1.002)
State of Emergency (SE)	20.181*** (1.471)	14.970*** (0.701)	8.339*** (0.700)	8.155*** (0.743)	7.045*** (0.983)
SC Announcement (Feb. 27)			0.141 (0.527)		
SE Announcement (Start, Apr. 7)			4.413*** (0.774)		
SE Announcement (Start, Apr. 16)			6.482*** (0.466)		
SE Announcement (End, May 14)			3.489*** (0.887)		
SE Announcement (End, May 21)			0.146 (0.518)		
SE Announcement (End, May 25)			-2.943*** (0.896)		
No. of New Infections Within Prefecture		1.808* (0.919)	2.960*** (0.604)	3.255*** (0.558)	2.234*** (0.361)
No. of New Infections Nationwide		2.644*** (0.245)	0.468** (0.216)		
Rain	3.408*** (0.561)	2.944*** (0.309)	2.638*** (0.238)	2.307*** (0.375)	0.873*** (0.235)
Weekend/Holiday	6.143*** (0.378)	6.175*** (0.369)	6.292*** (0.360)		
Obs.	8225	8225	8225	8225	8225
Adjusted *R*^2^	0.708	0.819	0.886	0.942	0.977
FEs	Prefecture	Prefecture	Prefecture	Prefecture	Prefecture
				Day	Day × Region
Weights	No. of smartphone users in each prefecture as of Jan 2020				

Notes: Figures in parentheses are cluster-robust standard errors.

*, **, and *** denote statistical significance at the 10%, 5% and 1% level, respectively. The *Rain* dummy takes 1 if the amount of precipitation in the prefectural capital was greater than 0, and takes 0 otherwise. The *Weekend/Holiday* dummy takes 1 for Saturdays, Sundays, and public holidays, and 0 otherwise. For the number of new infections within prefectures and nationwide, the inverse hyperbolic sine transforms ($\text{arcsinh}\left(x\right)=\ln\left(x+\sqrt{x^{2}+1}\right)$) were used.

As highlighted by Coibion et al. (2020) and others, the increase in the number of infections not only triggered government responses, such as the declaration of a state of emergency in Japan’s case, it also had the effect of increasing people’s fear of infection and led them to voluntarily refrain from leaving their homes. The increase in the stay-at-home measure in specification (1) thus may be due to an increase in the number of infections rather than government measures. Therefore, in specification (2), we added the number of new infections within the prefecture and the number of new infections nationwide as explanatory variables. Adding those variables reduces the coefficients on the *School Closure* and *State of Emergency* dummies from specification (1). However, the coefficients on all dummy variables remain significantly different from zero, indicating that the school closures and the state of emergency still had a statistically significant effect on the stay-at-home measure even after controlling for the number of infections.

Next, in specification (3), in order to control for changes in the stay-at-home measure due to the announcement effect of school closures and the state of emergency, we added a dummy for the announcement of school closures by the government on February 27, while for the state of emergency we added the two dummies for the announcement of the start of the state of emergency and the three dummies for the announcement of the lifting of the state of emergency. For instance, the *State of Emergency Announcement* (*Start, Apr. 7*) dummy takes 1 for all prefectures from April 8, the day after the announcement, and if the coefficient is positive, this indicates that the state of emergency announcement on April 7 raised the stay-at-home measure regardless of whether the state of emergency applied to a particular prefecture.

Looking at the effects of the closure of schools based on the results for specification (3), the coefficient on the *School Closure* dummy is around 9.3 and significantly different from zero. However, the coefficient on the *School Closure Announcement* dummy is small and not significantly different from zero. This shows that there was a large intervention effect that exceeded the information effect. Next, looking at the effect of the state of emergency declaration, the coefficient on the *State of Emergency* dummy is 8.3, showing that the intervention effect of the state of emergency had more or less the same size as the closure of schools. Moreover, the coefficients on the two dummies for the announcement of the start of the state of emergency are both positive and significant, and the effect was to increase the stay-at-home measure by 10.9 percentage points in total. On the other hand, the sum of all coefficients for the announcement of the lifting of the state of emergency is close to zero. The above results show that the declaration of the state of emergency had the effect of raising the stay-at-home measure through both the intervention effect and the information effect.

Next, specification (4) includes time fixed effects. Time fixed effects capture factors that have the same effect for all prefectures. They capture the effect that people refrain from leaving their homes in response to various other types of information about the pandemic, not only of government announcements about school closures and the state of emergency. The coefficients on both the *School Closure* dummy and the *State of Emergency* dummy are somewhat smaller than in specification (3) but remain statistically significant.

**Fig 7:**
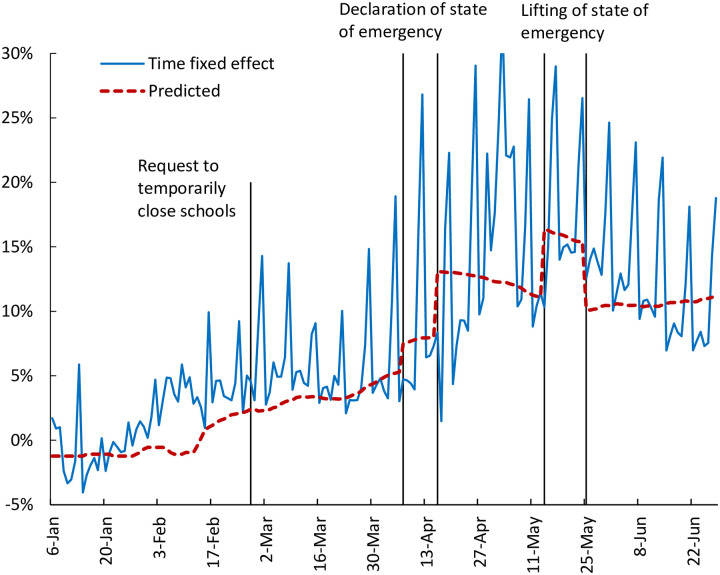
Estimates of time fixed effects. The blue line shows the coefficients on the time dummies. The dotted red line shows the fitted values obtained as follows: (1) we regress the estimated time fixed effects on the number of infections nationwide, the declaration of the state of emergency (April 7 and 16), the lifting of the state of emergency (May 14 and May 25), and *Weekend/Holiday* dummy; (2) we set the coefficient on the *Weekend/Holiday* dummy to zero in order to eliminate periodical fluctuations due to the weekend/holiday effect.

The blue line in Fig 7 shows the coefficients on the time dummies obtained in this estimation. The time fixed effects show two jumps in April. The first jump corresponds to the declaration of the state of emergency for seven prefectures on April 7, while the second one corresponds to the expansion of the state of emergency to all prefectures on April 16. The dotted red line in Fig 7 shows the predicted values obtained by regressing the time fixed effects estimated in specification (4) on the number of infections nationwide, the declaration of the state of emergency (April 7 and 16), the lifting of the state of emergency (May 14 and May 25), and the *Weekend/Holiday* dummy. Looking at the results, although the predicted value captures changes in the time fixed effect, and much of the change in the time fixed effect is explained by variables such as the announcements regarding the state of emergency, developments in the predicted value during some periods differ from the actual time fixed effect. For instance, in early February, there is no change in the dotted red line despite the increase in the time fixed effect. The latter likely reflects the fact that the Diamond Princess, a cruise ship with confirmed cases of coronavirus on board called at Yokohama Port on February 3 and was put under quarantine on February 5, which was widely reported in the media and gained public attention. It is possible that this news led people in all prefectures to refrain from leaving their homes. In contrast, there is no clear change in the estimated time fixed effects on March 24, when it was announced that the Tokyo Olympic Games scheduled for the summer of 2020 were to be postponed, suggesting that this had already been anticipated.

In specifications (3) and (4), we assumed that residents in all prefectures reacted to government announcements in the same manner. However, people’s reaction may differ depending on where they live. Therefore, in specification (5), we divide Japan into seven regions (Tohoku, Kanto, Kinki, Chubu, Chugoku, Shikoku, and Kyushu) and, by specifying the time fixed effect as Day FE × Region, allow for the possibility that the time fixed effect may differ across regions. Looking at the estimation results, the coefficient on *School Closure* is 4.8, while the coefficient on *State of Emergency* is 7.0, which shows that the coefficient on *School Closure* has dropped significantly. However, the coefficients for both *School Closure* and *State of Emergency* continue to be positive and significant. Further, as a robustness check, we conducted the same regressions as in Table 1 using observations from 21 prefectures only, consisting of the seven prefectures in which the state of emergency was declared on April 7 and the 14 prefectures adjacent to these seven prefectures. The purpose of these additional regressions is to more precisely identify the intervention effect associated with the declaration of the state of emergency on April 7 by restricting the comparison to neighboring prefectures that are likely to be more similar to the seven prefectures than other prefectures. We find that the regression results are essentially the same as in Table 1.

The estimations in Table 1 took into account that the constant for weekends/holidays and weekdays may differ by including a *Weekend/Holiday* dummy. However, it is possible that not only the constant but also the coefficients on the independent variables may differ between weekends/holidays and weekdays. Whether people leave their homes on weekdays to a large extent depends on how their workplace or school responds to government requests. On the other hand, with regard to going out on weekends/holidays, it is individuals who make the decision, and hence the response depends on how individuals react to requests from the government. Ref [15] conducts separate estimations for weekdays/holidays to show that the intervention effect is larger for weekdays than weekends/holidays. They interpret this as resulting from firms actively promoting the shortening of business hours and the shift to work from home in response to the government’s requests. On the other hand, they show that the information effect is larger for weekends/holidays than weekdays. They interpret this as indicating that the information effect is driven by consumers, not by firms or workers.

Let us compare the results of specification (5) with previous studies on the United States. Ref [9] found that a shelter-in-place (S-I-P) order in a certain county reduced the number of customers visiting retail stores in that county by 7.6%. This is the intervention effect of S-I-P orders in the United States. To compare this with our results, we convert our estimate as follows. As the stay-at-home measure immediately before the state of emergency was declared was 0.17, the level of outings, relative in January 2020 before the outbreak of the pandemic, was 0.83. The coefficient on the *State of Emergency* dummy in specification (5) of 7.0 means that the level of outings dropped to 0.76 due to the intervention effect of the emergency declaration (i.e., 0.83-0.07 = 0.76), and the rate of change in outings is -8.5%. Thus, although the figures are not directly comparable, since ref [9] measure the reduction in outings using the number of customers visiting stores, the order of magnitude of their result and ours—a decline of 7.6% due to S-I-P orders in the United Sates and of 8.5% due to the declaration of the state of emergency in Japan—is quite similar. Interestingly, therefore, the intervention by the Japanese government in the form of a “request” and the legally binding lockdowns in the United States had more or less the same intervention effect.

Next, let us compare the coefficient on new infections within the prefecture in specification (5) with results for the United States. The coefficient on the number of new infections within the prefecture is 2.2, and, based on the level of the stay-at-home measure just before the state of emergency was declared, an increase in the number of new infections in the prefecture by 1% led to a reduction in outings of 0.027%. The level of outings immediately before the state of emergency was declared was 0.83. The coefficient on new infections within the prefecture in specification (5) of 2.2 means that the level of outings dropped by 0.00022 in response to a 1% increase in the number of new infections, implying that the rate of change in outings is -0.027%. On the other hand, ref [9] showed that an increase in the cumulative number of deaths from COVID-19 by 1% reduced the number of consumer visits by 0.03%. Although the figures cannot be directly compared, since one refers to the number of infections while the other refers to the number of deaths, they suggest that people in Japan and the United States changed their behavior more or less the same way in response to information on the spread of COVID infections.

How should the results of positive and statistically significant coefficients on the *School Closure* and *State of Emergency* dummies be interpreted? Regarding school closures, Prime Minister Abe proposed that schools be closed and requested local governments such as prefectures and municipalities, which have jurisdiction over schools, to do so. Local governments accepted this request, and four days after Prime Minister Abe’s proposal, schools actually closed. This sequence of events suggests that Prime Minister Abe’s request had compelling force on local governments. Presumably, following the decisions by local governments, students (and their parents) started to refrain from leaving their homes. The above-mentioned results that school closures had an intervention effect support this.

Turning to the state of emergency, in contrast with the lockdowns in China, the United States, and Europe, Japan’s state of emergency was not legally binding. There is no rational reason for people to follow “requests and instructions” without penalties such as fines or arrests, and another explanation why the declaration of the state of emergency had the effect it did is needed. One possibility is that the government’s request triggered a change in strategic relationships among companies. For example, a major issue when a firm considers whether to shorten working hours or switch to working from home is how other firms that it does business with react. If business partners do not shorten working hours or allow their employees to work from home, it is not desirable for the firm to switch on its own. And if all firms think like this, no firm will switch. However, if a firm’s business partners switch to shorter working hours or to working from home, it is desirable for the firm to also switch. If this virtuous cycle is created, all firms will shorten their business hours and/or switch to working from home and, as a result, people will refrain from going out. It is possible that the government’s “request” triggered a change in expectations about how other firms will respond, which may have led to coordinated restraint from going out. Another possibility is that the government’s request triggered an increase in social pressure to conform with restraint from going out. This is symbolized, for example, by the emergence of a “self-restraint police” (“virus vigilantes”) that look for and criticize people who are out and about.

### 5.2 Decomposition of changes in the stay-at-home measure

**Fig 8:**
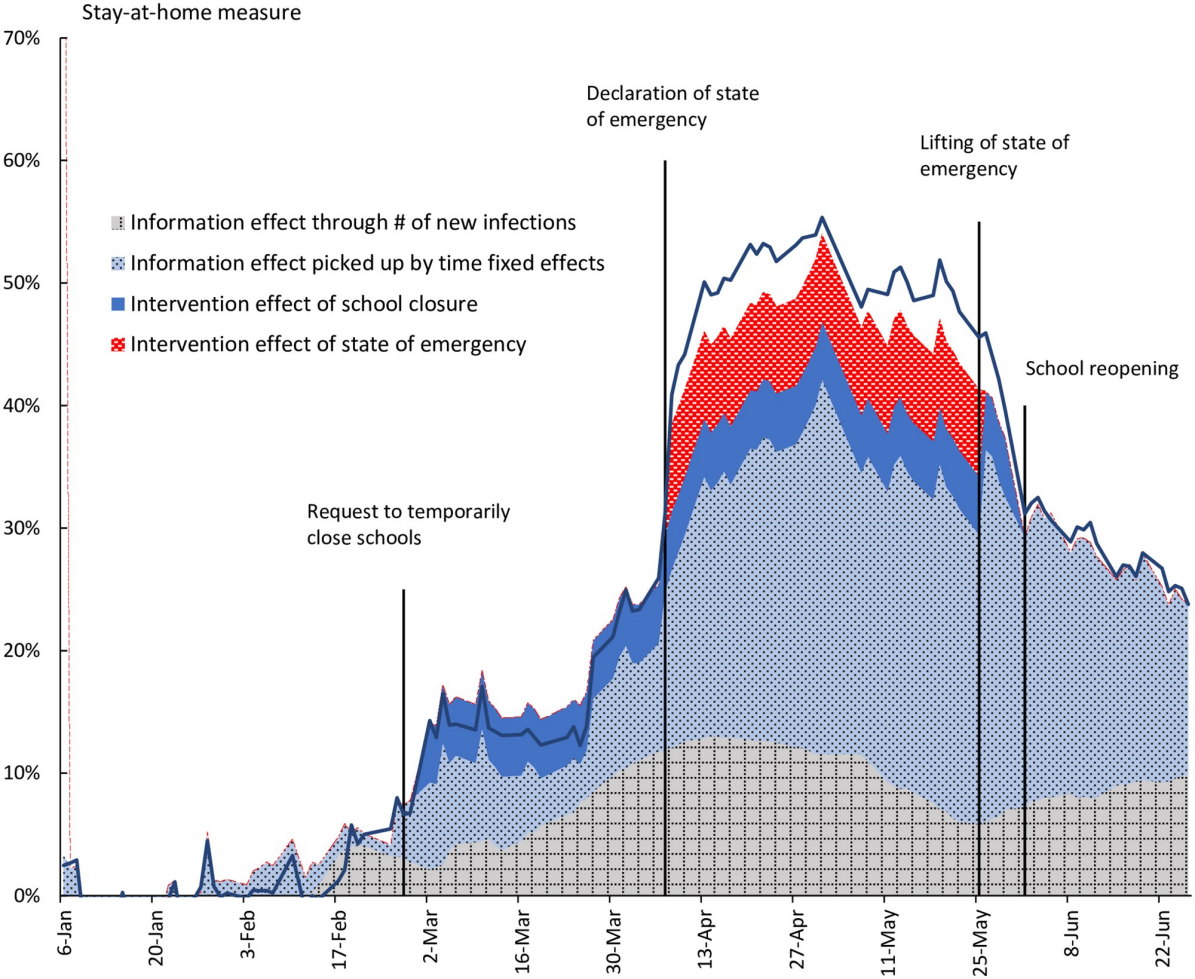
Decomposition of changes in the stay-at-home measure for Tokyo. Changes in the stay-at-home measure for Tokyo are decomposed into the intervention and the information effects using the estimation results from specification (5) in Table 1. To eliminate seasonal fluctuations, only weekday observations are used for the stay-at-home measure.

Fig 8 presents a decomposition of changes in the stay-at-home measure for Tokyo using the estimation results from specification (5) in Table 1. During the period examined here, the stay-at-home measure for Tokyo peaked at 55% on May 1, and dividing the increase from January into the contribution of the various components shows that the intervention effect of school closures contributed 5 percentage points and the intervention effect of the state of emergency contributed 7 percentage points, for a combined intervention effect of 12 percentage points. On the other hand, the contribution of the number of new infections within Tokyo prefecture was 12 percentage points, while the contribution of the time fixed effect was 30 percentage points, for a combined information effect of 42 percentage points. Thus, the intervention effect contributed about a quarter and the information effect about three quarters to the reduction in outings, indicating that the dominant channel for changes in behavior was the information effect.

## 6 Summary and policy implications

In China, the United States, and Europe, legally binding interventions such as lockdowns have been used to prevent people from leaving their homes. On the other hand, in Japan, such intervention took the form of a government “request” calling on people to refrain from going out. At the time, there were many in Japan who were concerned that such a “request” would not have a sufficient effect. However, the analysis in this study has shown that the Japanese government’s declaration of a state of emergency to a certain extent was successful in changing people’s behavior. Specifically, in prefectures where a state of emergency was declared, outings fell by 8.5%. According to [9], in the United States, the number of customers visiting retail stores decreased by 7.6% in counties that had imposed a lockdown. Thus, interestingly, the effects of government intervention were similar in Japan and the United States.

What does this finding mean? First, both the compulsory lockdown in the United States and the voluntary lockdown in Japan had a substantial impact on people’s mobility. In both countries, the movement of people decreased substantially compared to normal times. However, the lockdowns were responsible only for part for this reduction. The remainder of the reduction was due to the fact that these measures increased people’s awareness of the seriousness of the pandemic, for example through government announcements and the release of the number of infections and deaths; in other words, the remainder was due to the information accompanying these measures, which led people to voluntarily refrain from going out. Thus, the lesson of the experience both in Japan and the United States is that in order to contain infections, it is necessary to provide people with correct information in a timely manner and to encourage voluntary changes in behavior.

Second, if government intervention is needed, what is preferable: a compulsory lockdown or voluntary lockdown? The advantage of a compulsory lockdown is that its effects can be predicted to some extent, and uniform changes in behavior can be expected for a wide range of people (though not all). On the other hand, from citizens’ point of view, a compulsory lockdown imposes severe restrictions on their personal freedom: to avoid being penalized with a fine or arrest, people have to respond in a uniform manner regardless of their individual circumstances. By contrast, in a voluntary lockdown, there is room for each citizen to decide whether or not to comply with the request, based on their own personal circumstances. On the other hand, though, the effect is uncertain. Whether a similar request for voluntary lockdown would be effective in Japan in the future or in other countries such as the United States or in Europe is unclear. While this study suggests that the government’s request may have provided the impetus for cooperation to avoid the spread of COVID-19 infections, further analysis is needed.
